# A small molecule improves diabetes in mice expressing human islet amyloid polypeptide

**DOI:** 10.1080/19382014.2022.2163829

**Published:** 2023-01-12

**Authors:** Vriti Bhagat, C. Bruce Verchere

**Affiliations:** a Department of Pathology and Laboratory Medicine, The University of British Columbia, Vancouver, BC, Canada; b BC Children’s Hospital Research Institute, Vancouver, BC, Canada; c Centre for Molecular Medicine and Therapeutics, The University of British Columbia, Vancouver, BC, Canada; d Department of Surgery, The University of British Columbia, Vancouver, BC, Canada

**Keywords:** Islets, beta cells, amyloid, islet amyloid polypeptide, amylin, small molecule, autophagy

## Abstract

In recent years, the number of studies on islet and beta cell autophagy have substantially increased due to growing interest in the role of autophagy in maintaining cellular homeostasis in diabetes. In type 2 diabetes, human islet amyloid polypeptide (hIAPP) aggregates to form higher structure oligomers and fibrils that are toxic to beta cells and induce islet inflammation. The primary mechanism of oligomer and fibril clearance in beta cells is through the autophagic pathway, a process that is impaired in type 2 diabetes. Thus, toxic oligomeric and fibrillar forms of hIAPP accumulate in type 2 diabetic islets. Recently, Kim *et al*. characterized the ability of a small molecule autophagy enhancer, MSL-7, to clear hIAPP oligomers in mice expressing hIAPP. Herein, we outline the primary findings of the study, limitations, and future directions to further investigate the therapeutic potential of autophagy enhancers to treat diabetes.

## Graphical Abstract

**Figure:**
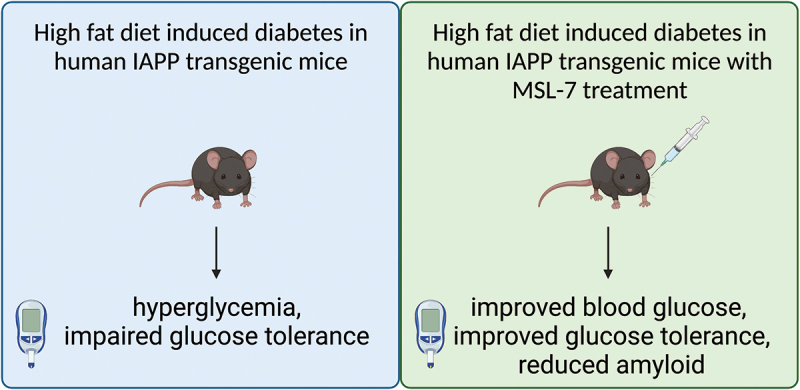

## Article

Diabetes is one of the fastest growing global health burdens with over half a billion individuals diagnosed with diabetes worldwide.^1^ A characteristic of diabetes is impairment of normal cellular processes in beta cells including autophagy, a form of cell death. In nearly all animal cells, the autophagic pathway degrades cellular molecules that have improper function. Basal levels of autophagy are necessary to prevent the accumulation of misfolded proteins that are harmful to cells.^2^ In type 2 diabetes (T2D), the ability to clear misfolded proteins through autophagy is diminished. Writing in *Nature Communications*, Kim *et al*.^3^ report on the ability of a small molecule to increase autophagic flux and improve glucose tolerance in a mouse model of T2D.

Dysregulation of blood sugar levels and impaired glucose tolerance are hallmarks of T2D. Pancreatic beta cells not only produce insulin, which is critical for regulating blood glucose, but also another peptide hormone called islet amyloid polypeptide (IAPP). IAPP is co-secreted with insulin from beta cells and functions synergistically with insulin to regulate blood glucose following a meal.^4^

In T2D, IAPP monomers aggregate to form higher structure oligomers and fibrils that disrupt cellular processes. Indeed, the presence of IAPP fibrils has been identified in greater than 90% of patients with T2D.^5^ Because IAPP oligomers are thought to be preferentially cleared in beta cells via the autophagic pathway,^2^ and autophagy is impaired in T2D (as well as in type 1 diabetes),^6^ toxic IAPP oligomers likely accumulate in the islets of persons with T2D. Thus, a question in the field remains whether increasing autophagic flux may improve T2D health outcomes.

Kim *et al*.^3^ addressed this question by investigating the role of a small molecule autophagy enhancer as a potential therapeutic agent for diabetes. Small molecule enhancers are compounds of low molecular weight that amplify the normal function of their target. In a previous study, these researchers identified a chemically modified derivative (MSL-7) of the novel autophagy enhancer MSL [4-(4-fluorophenyl)sulfonyl-5-methylthio-2-phenyloxazole] as a potential drug candidate for diabetes.^7^ Here, the group investigated whether the small molecule MSL-7 is able to increase autophagy, reduce IAPP oligomers, and improve glucose tolerance.

**Figure 1.**
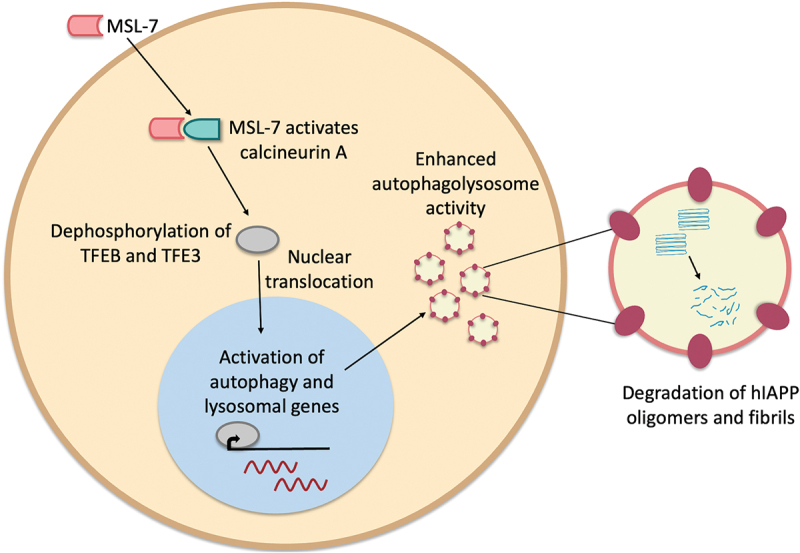
**Caption: Mechanism of action of the small molecule autophagy enhancer, MSL-7**. MSL-7 enters cells from the extracellular environment to activate the phosphatase calcineurin A in the cytoplasm. Calcineurin A dephosphorylates two regulators of autophagy and lysosomal biogenesis, the transcription factors TFEB and TFE3. TFEB and TFE3 translocate to the nucleus and activate the expression of autophagy and lysosomal genes. This results in enhanced autophagolysosome activity. Toxic human IAPP oligomers and fibrils are degraded by enzymes in the autophagolysosomes. Figure of a cell. A pink shape representing the small molecule MSL-7 enters the cell from the extracellular environment shown with an arrow. The pink shape representing MSL-7 is shown beside another turquoise shape representing calcineurin A to depict calcineurin A activation. An arrow then points to a gray oval representing the transcription factors TFEB and TFE3, which is then shown to enter the nucleus of the cell by another arrow. In the nucleus is the gray oval shape representing the transcription factor on a black line with a short arrow pointing to the right to depict transcription of autophagy and lysosomal genes. Nuclear mRNAs are depicted with red wavy lines. An arrow points to autophagolysosomes in the cytoplasm. One autophagolysosome is enlarged to the right of the cell to show the contents. The enlarged autophagolysosome shows long blue wavy lines clustered together representing human IAPP oligomers and fibrils, and smaller blue lines representing degraded human IAPP oligomers and fibrils.

First, Kim and colleagues assessed the ability of MSL-7 to induce autophagy (Figure 1) in a rat beta cell line, INS-1 cells. They observed an increase in nuclear translocation of two regulators of autophagy and lysosomal biogenesis (TFEB and TFE3), and an increase in the number of autophagolysosomes, an intracellular compartment that degrades cellular components through autophagy. Accordingly, autophagy and lysosomal genes were upregulated, demonstrating that MSL-7 increases autophagy in a rodent beta cell line.

To investigate oligomer clearance by MSL-7, the authors then modified INS-1 cells to express human IAPP (hIAPP), since rodent IAPP does not aggregate or induce beta-cell toxicity. hIAPP dimers accumulate in hIAPP-expressing INS-1 cells, and this was reversed by addition of the autophagy enhancer MSL-7. These data support the idea that hIAPP oligomer clearance in beta cells is mediated by autophagy and can be increased by small molecule autophagy enhancers. Notably, when the aforementioned experiments were repeated in monkey islets, the human beta cell line 1.1B4, and human induced pluripotent stem cell derived islets, similar results were observed, highlighting the consistency of the effect of MSL-7.

Next, to study the role of MSL-7 *in vivo*, Kim and colleagues induced diabetes by high fat diet in mice with beta-cell expression of hIAPP, and simultaneously treated one group with MSL-7. After 8 weeks, the group administered MSL-7 displayed decreased non-fasting and fasting blood glucose levels, as well as improved glucose tolerance. Although serum insulin levels were not significantly different between hIAPP transgenic mice treated with MSL-7 or vehicle control, insulinogenic index and oxygen consumption rate were significantly improved. Taken together, these results indicate that MSL-7 protects hIAPP transgenic mice from hyperglycemia and improves beta cell function.

Interestingly, immunostaining using the anti-oligomer A11 antibody suggested that treatment with MSL-7 reduced hIAPP oligomers in islets of hIAPP transgenic mice. Further, pancreas sections of MSL-7 treated hIAPP mice displayed increased nuclear translocation of TFEB and TFE3. When *Tfeb* was deleted from beta cells, these *in vivo* findings were significantly diminished, illustrating that MSL-7 enhancement of hIAPP oligomer autophagy *in vivo* is mediated via activation of TFEB and TFE3.

These intriguing results support previous research that has demonstrated impairment of beta-cell autophagy in diabetes.^2^ Kim and colleagues provide new evidence that increasing autophagic flux may attenuate beta cell dysfunction in diabetes. Importantly, these data suggest that MSL-7 or other autophagy enhancers could be potential therapeutic agents to treat T2D.

In this study, the authors induced diabetes with a high fat diet and began treatment with MSL-7 at the same time. A future avenue worth exploring is whether MSL-7 is capable of clearing IAPP oligomers in mice that were already rendered diabetic. Typically, T2D is not diagnosed and treated in humans until individuals are already diabetic, a point at which IAPP aggregation and toxicity has likely already begun. Treating hIAPP transgenic mice that are already diabetic with MSL-7 might better resemble the intervention timeline in humans.

This study demonstrates a unique strategy to potentially treat diabetes, but some limitations to the methodology used by the authors are evident. The authors used beta cell lines, monkey islets, and mouse islets, which do not represent human physiology as well as primary human beta cells.^8,9^ Furthermore, the mouse model used overexpresses hIAPP, with higher expression and aggregation than human islets, and rapid amyloid formation. An alternative approach to study the potential therapeutic benefit of MSL-7 with greater relevance to human biology might be to transplant human islets from organ donors into mice, to determine whether MSL-7 can clear hIAPP oligomers found in human islets in an *in vivo* environment.

Oligomerization of hIAPP is a distinct characteristic of T2D and is associated with autophagic dysregulation. Kim *et al*. demonstrate the ability of a small molecule autophagy enhancer to clear hIAPP oligomers from islets. This finding merits further investigation into the possibility of using autophagy enhancers as therapeutic agents to treat diabetes.
